# Bone involvement in non-congenital syphilis

**DOI:** 10.7705/biomedica.6603

**Published:** 2023-06-30

**Authors:** Daniela Trujillo, Carlos Andrés Agudelo, Andrés Chavarriaga, Pablo Villa, Alejandro Vélez, Ricardo Cardona, Alicia Hidrón

**Affiliations:** 1 Programa de Enfermedades Infecciosas, Escuela de Ciencias de la Salud, Universidad Pontificia Bolivariana, Medellín, Colombia Universidad Pontificia Bolivariana Programa de Enfermedades Infecciosas Escuela de Ciencias de la Salud Universidad Pontificia Bolivariana Medellín Colombia; 2 Sección de Reumatología, Clínica CES, Medellín, Colombia Sección de Reumatología Clínica CES Medellín Colombia; 3 Sección de Enfermedades Infecciosas, Hospital Pablo Tobón Uribe, Medellín, Colombia Sección de Enfermedades Infecciosas Hospital Pablo Tobón Uribe Medellín Colombia; 4 Sección de Patología, Hospital Pablo Tobón Uribe, Medellín, Colombia Sección de Patología Hospital Pablo Tobón Uribe Medellín Colombia

**Keywords:** Syphilis, bone and bones, bone neoplasms, neurosyphilis, HIV, sífilis, huesos, neoplasias óseas, neurosífilis, VIH

## Abstract

We documented two stages of bone involvement due to syphilis in two adult patients infected with human immunodeficiency virus. Bony lesions of secondary versus tertiary syphilis cannot be differentiated on clinical or radiologic grounds alone. Given the rarity of this clinical presentation, there is no consensus on treatment duration and related outcomes.

## Resumen

Se describen dos etapas de compromiso óseo por sífilis en dos pacientes adultos infectados por el virus de la inmunodeficiencia humana. Las lesiones óseas de la sífilis secundaria y de la sífilis terciaria no se pueden diferenciar únicamente por características clínicas o radiológicas. Dada la rareza de esta presentación clínica, no hay consenso sobre la duración del tratamiento y los resultados relacionados.

The introduction of penicillin changed the natural history of syphilis: The wide array of clinical manifestations reported in the pre-antibiotic era was limited to a few case reports thereafter. However, there has been a resurgence of previously uncommon clinical presentations owing to the HIV epidemic. Here, we compare the type of bone involvement in secondary and tertiary syphilis in two HIV-infected patients.

## Case 1

A 27-year-old HIV-infected male on antiretroviral treatment (ARV) with a CD4 count of 297 cells/mm^3^ and an undetectable viral load (<20 copies/ml) presented to the emergency unit with a three-week history of progressive frontal headache without any response to analgesics. He was diagnosed with secondary syphilis three months before and underwent incomplete treatment due to a severe anaphylactic reaction to penicillin.

**Figure 1:**
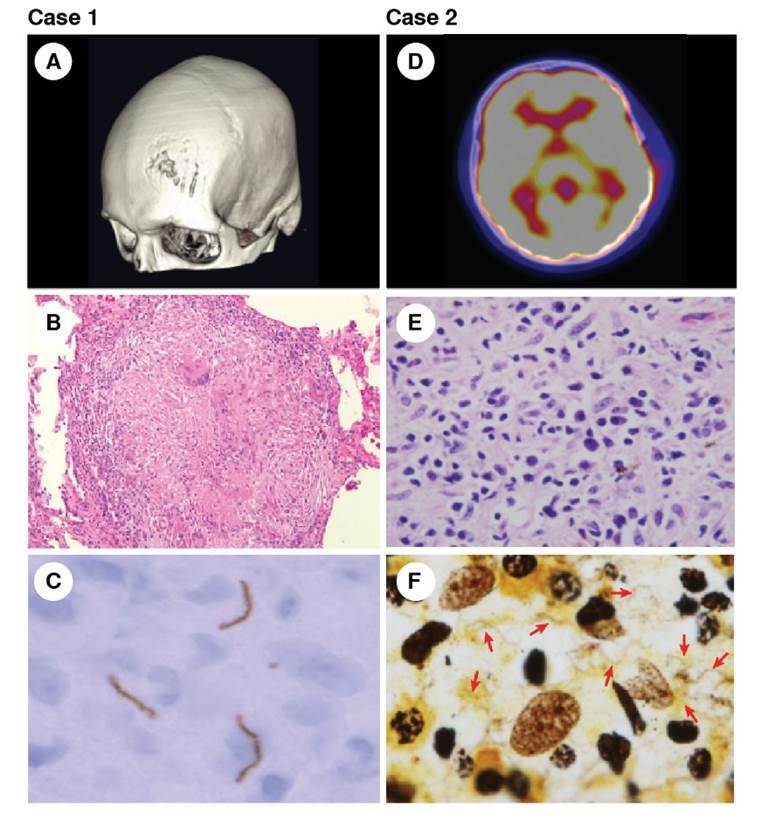
Left column: Case 1 - Tertiary syphilis. (A) Three-dimensional reconstruction of the skull computed tomography showing the bone lesion in the frontal bone. (B) Syphilitic gumma with chronic granulomatous infiltrate, hematoxylin eosin, 10X. (C) Immunochemistry of the lytic bone lesion showing spirochetes in the tissue. Right column: Case 2 - Secondary syphilis. (D) PET scan revealing lytic lesions with increased metabolism on the frontal and temporal bones. (E) Lymphoplasmocytic infiltrate in the bone biopsy, hematoxylin eosin, 100X. (F) Warthin-Starry stain revealing the presence of numerous spirochetes, 100X

On the physical exam, a 3 x 3 cm non-painful nodular lesion on his forehead and erythematous macules in his palms were observed. The Rapid Plasma Regain test (RPR) (1:32 dilutions) and the confirmatory test (Fluorescent Treponemal Antibody Absorption - FTA-ABS) were positive. Cerebrospinal fluid (CSF) studies revealed a glucose of 45 mg/dl, 20 white blood cells/ml, 61 mg/ dl of protein and a positive VDRL (1:8 dilutions). Computed tomography (CT) of the head revealed a lytic lesion in the left frontal bone (figure 1A).

The bone lesion biopsy revealed a chronic granulomatous process with no necrosis or perivascular lymphoplasmocytic infiltrate. Warthin Starry stain was negative, but immunohistochemical staining was positive for spirochetes (figure 1B). The lesion was cataloged as syphilitic gumma based on the chronic granulomatous infiltrate and the presence of spirochetes (figure 1C).

After completing the desensitization protocol, the patient started treatment with 4 x 10^6^ units of intravenous penicillin every four hours. The patient completed a 14-day course and at the 6-month follow-up, resolution of the headache and all lesions was documented.

## Case 2

A healthy 40-year-old male presented to the emergency unit with a 20- day history of pain in the right side of his rib cage, more intense at night, and weight loss.

In the physical exam, a painful nodular lesion on the right sixth rib was detected. A chest computed tomography (CT) showed a lytic lesion on the sixth rib. A bone scan and positron emission tomography (PET) revealed additional lytic lesions with increased metabolism on the frontal and temporal bones (figure 1D). A bone biopsy was performed.

During his hospital stay, the patient reported a new-onset headache, pain in the nodular lesion in his forehead, and a diffuse erythematous maculopapular rash on his palms and soles.

The bone biopsy showed a lymphoplasmocytic infiltrate without necrosis or granuloma formation (figure 1E). The Warthin Starry stain revealed the presence of numerous spirochetes (figure 1F). With this result, treponemal and non-treponemal tests were ordered, both with positive results (Venereal Disease Research Laboratory - VDRL 1:128 dilutions). Cerebrospinal fluid (CSF) studies revealed glucose levels of 57 mg/dl, 19 WBC/ml, and 36 mg/dL of protein; the CSF VDRL was negative. The ophthalmologic evaluation found vitritis and optic neuritis in the left eye. The HIV test was positive with a viral load of 74,534 copies/mL, and the CD4 count was 476 cells/ml.

The patient’s diagnosis was secondary syphilis with cutaneous involvement and neurosyphilis with ocular involvement. The patient started treatment with 4 x 10^6^ units of intravenous penicillin every four hours for 14 days and ARV. Three months later, the lesions were no longer painful or clinically appreciable, the headache had resolved, and the ocular lesions had disappeared. Serum VDRL was positive but only at a 1:2 dilution and CSF parameters were all normalized.

In a literature review from 1946 to 2022, we found 39 published cases of bone involvement in non-congenital syphilis (table 1). Most of these cases were reported as secondary syphilis (34 cases, 87%), only four cases were tertiary syphilis, and one was considered as late latent syphilis. The mean age at presentation was 34 years (20-60 years) and only 13% of patients were women; 30% (10) of cases were reported in HIV-infected patients with an average CD4 count of 367 cell/mm^3^ (130689 cell/mm^3^). The most frequent radiographic finding was lytic lesions, mainly in the frontal and parietal bones (18 cases, 47%), followed by the tibia (45.9%).

**Table 1:** Published cases of bone involvement in non-congenital syphilis and the two cases presented here

Authors (Year)	Age sex	HIV status	Radiographic examination	Localization of lesions	Symptoms and signs	Stage of syphilis	Treatment	Length of treatment	Response
Lefkovits AM, *et al*. (1946)	22/M	No	Nodular lesion and erosion of the outer layer	Skull	Headache, alopecia, generalized lymphadeno-pathy	Secondary	Procaine penicillin G	Two and a half weeks	Decreased in size at three months
Kellock IA, *et al*. (1956)	47/M	No	Mixed lytic and sclerotic lesion	Skull, tibia, humerus, femur, elbow	Bone pain, weight loss, headache	Secondary	Benzathine penicillin G	Ten days	Improvement of images at five months
Bauer MF, *et al*. (1967)	26/W	No	Osteolytic lesion in frontal bone	Frontal bone	Headache, maculopapular rash	Secondary	Procaine penicillin G	Ten days	Partial resolution at three months
Parker JDJ. (1972)	35/M	No	Periosteal reaction	Tibias	Leg pain with paraesthesia of the ankles	Secondary	Cephalori-dine	Two weeks	Almost complete resolution of images at 11 months
Tigh RR, *et al*. (1976)	20/M	No	Multiple areas of increased uptakea	Skull, sternum, and clavicle	Headache, myalgia, maculo-papular rash, cervical lymphadenopathy	Secondary	Benzathine penicillin G	Ten days	Loss at follow-up
Longstreth P, *et al*. (1976)	30/M	No	Bone resorption of the distal clavicle	Clavicle	Headache, shoulder pain, alopecia, splenomegaly	Secondary	Benzathine penicillin G	Three weeks	Recalcification at one month
Erlich R, *et al*. (1976)	25/M	No	Multiple lytic lesions	Tibia, fibula, ulna, radius	Headache, myalgia, ankle pain, maculopapular rash	Secondary	Benzathine penicillin G	Two and a half weeks	Imaging resolution at three months
Dismukes WE, *et al*. (1976)	32/M	No	Multiple osteolytic lesions	Clavicle, frontal and parietal bone	Headache, fever, weight loss, generalized lymphadenopathy, maculopapular rash	Secondary	Procaine penicillin G	Ten days	Asymptomatic
Shore RN, *et al*. (1977)	37/M	No	Multiple lytic lesions	Tibia, fibula, radius, and ulna bilaterally	Pain in leg, ankles and arms, maculo-papular rash	Secondary	Procaine penicillin G	Ten days	--
Graudal C, *et al*. (1981)	42/M	No	Osteitis changes^a^	Tibia	Left leg pain, fever, sore throat, weight lost	Secondary	Benzathine penicillin G	Ten days	Imaging resolution after treatment
Petersen LR, *et al*. (1983)	48/M	No	Diffuse increased activity^a^	Ribs, long bones, and spine	Leg and back pain, headache, papular rash	Secondary	Parenteral penicillin G	Ten days	Asymptomatic at two months
Hansen K, *et al*. (1984)	31/M	No	Multiple areas of increased uptake^a^	Parietal, occipital, maxillary, mandibular, ribs, humerus, femur	Headache, fever	Secondary	Benzathine penicillin G	Five months	50% reduction in activity at nine months
Veerapen K, *et al*. (1985)	35/W	No	Increased uptake^a^	Frontal bone and tibia	Neck and leg pain	Secondary	Procaine penicillin G	Ten days	No changes in the images at six months
Meier JL, *et al*. (1986)	37/M	No	Increased activity^a^	Tibias	Fever, leg pain, headache	Early syphilis	Penicillin	Ten days	Asymptomatic at one year
Rodriguez S, *et al*. (1988)	39/M	No	Dense bony sclerosis	T10, L1 and L5 vertebral bodies	Weight loss	Tertiary	Procaine penicillin G	One month	Asymptomatic at three months
Ollé-Goig JE, *et al*. (1988)	32/W	No	Multiple diffuse intracortical destructive lesions	Tibias, fibulas	Leg pain, weight loss, headache	Secondary	Procaine penicillin G	Ten days	Asymptomatic at six months
	21/M	No	Osteolytic lesión	Frontal and parietal bone	Asthenia, headache, generalized lymphadenopathy, maculopapular rash	Secondary	Benzathine penicillin G	Ten days	Asymptomatic at two months
Middleton S, *et al*. (1990)	31/M	No	Increased uptake^a^	Tibias, ulnas, and frontal bone	Leg and forearm pain	Secondary	Benetha- mine penicillin	Ten days	Imaging resolution at four months
Kastner RJ, *et al*. (1994)	25/M	No	Increased uptake^a^	Ulna, skull, and radius	Maculo-papular rash, generalized lymphadenopathy	Secondary	Procaine penicillin G	Two weeks	Markedly diminished uptake at 30 days
Chung KY, *et al*. (1994)	29/W	No	Round osteolytic lesions	Frontal, temporal, and parietal bones	Headache, maculopapular rash	Secondary	Benzathine penicillin G	Three weeks	
Rademacher	27/W	No	Periosteal elevation	Tibias, calcaneus	Headache, fever,	Secondary	Intrave-nous	Two weeks	Lost at follow-up
SE, et al. (1996)	34/M	Yes	with periostitis Cortical thickeninga	Tibias	ulcerative rash, and shin and heel pain Fever, chills, night sweats, weight loss, painful skin lesions		penicillin G Intrave-nous penicillin G then benzathine penicillin	One month	Asymptomatic at nine months
Gurland IA, et al. (2001)	20/M	Yes	Multiple lytic lesions	Skull	Painful nodules	Secondary	Procaine penicillin G	Three weeks	--
Coyne K, et al. (2006)	36/M	Yes	Periostitis	Humerus, femur, tibia, fibula, and skull	Headache, fever, and sweats, maculopapular rash	Secondary	Benzathine penicillin G	Two weeks	Imaging resolution at three months
Huang I, et al. (2007)	40/M	Yes	Multiple lytic lesions, “worm eaten” appearance	Frontal and parietal bone	Headache	Secondary	Penicillin	One and a half months	Asymptomatic resolution at 1.5 months
Kandelaki G, et al. (2007)	20/M	Yes	Destructive lesion	Sternal bone	Painful lump on chest and maculo-papular rash	Secondary	Procaine penicillin G	Two weeks	Imaging resolution at six weeks
Denes E. (2009)	37/M	Yes	Increased uptakea	Tibia, radius, and skull	Leg pain, loss of appetite and weight, skin ulcerations	Secondary	Benzathine penicillin G	Six weeks	Asymptomatic at two months
Naraghi AM, et al. (2010)	64/M	Yes	Hetero-geneous activitya	Tibias, fibula, femur, skull, orbit	Leg pain	Early syphilis	Benzhatine penicillin G	Three weeks	Imaging resolution at three years
Samarkos M, et al. (2011)	25/M	Yes	Increased uptakea	Skull, ribs	Headache, maculopapular rash	Secondary	Intrave-nous penicillin G	-	Imaging resolution at three months
Liu Z-Y, et al. (2011)	62/M	No	Focal osteolytic lesions and onionlike periosteal reaction	Tibia and fibula	Leg pain	Late latent syphilis	Penicillin G	One and a half months	Imaging resolution at 1.5 months
Egan KM, *et al*. (2012)	41/M	No	Increased enhancement^a^	Skull	Headache and papular rash	Secondary	Procaine penicillin G	Two weeks	Asymptomatic at three months
Boix V, *et al.* (2013)	40/M	Yes	Osteolytic lesions and increased uptake^a^	Skull, humerus, and ulna	Fever, headache, weight loss, generalized lymphadenopathy	Secondary	Doxycy-cline and azithromycin	Four months	Imaging resolution at four months
Alraddadi B, *et al*. (2013)	32/M	-	Osteolytic lesion	Skull	Headache	Secondary	Intrave-nous penicillin G	Two weeks	Asymptomatic at two weeks
Park KH, *et al*. (2014)	41/M	No	Bone destruction and extra-skeletal soft tissue formation	Multiple ribs and L5 vertebra	Weight loss, maculopapular rash, and generalized lymphadenopathy	Secondary	Benzathine penicillin	Three weeks	Decreased uptake at six months
Bezaley S, *et al*. (2014)	20/M	No	Multiple osteolytic and sclerotic lesions with periosteal reaction	Tibia, 11th rib, parietal bone, acromioclavicular joint, and sacroiliac joint	Weight loss, leg, shoulder and rib pain, headache, maculopapular rash	Secondary	Penicillin	-	Asymptomatic at one month
Manríquez J, *et al*. (2014)	50/M	No	Increased uptake^a^	Tibia	Leg pain, maculopapular rash, generalized lymphadenopathy	Secondary	Benzathine penicillin G	Three weeks	-
Bai Y, *et al*. (2017)	44/M	No	Osteolytic lesion and new bone formation	Bodies of L4 and L5 vertebras	Low back pain, numbness below the knees, and inability to walk	Tertiary	Benzathine penicillin G	Three weeks	Imaging resolution at 12 months
Kamegai K, *et al*. (2022)	30/M	Yes	Osteolytic lesion	Sternum and ribs	Chest pain	Tertiary	Ceftriax-one	13 weeks	Magnetic resonance imaging (MRI) showed abnormal signals
Jankowska L, *et al*. (2022)	20/W		Osteolytic lesion	Clavicule	Skin lesions, uveitis, bone pain	Tertiary	Penicillin	14 weeks	Reduced tumor was observed

^a^ Bone scintigraphy

Twenty patients presented headache (51%), bone pain (48%) and maculopapular rash (17 patients, 43%). Among the five patients with tertiary or late latent syphilis, bone pain was the most frequent symptom (4/5 patients). Of the 39 patients, 35 (89%) were treated with intravenous penicillin, 17 (43%) with intramuscular procaine penicillin, and 14 (35%) with benzathine penicillin. The mean treatment duration was 24.6 days (10-150 days). No treatment failures were reported.

According to Resolution 8430 of 1993 of the Colombian *Ministerio de Salud*, the present report implied no risk: it used retrospective documentary research methods and information from medical records. The patients gave their informed consent, and it had the approval of the institutional ethics committee. There was no intervention or intentional modification of the patients’ biological, physiological, psychological, or social variables, and their dignity, well-being, and confidentiality were respected. Our research follows the bioethical principles of beneficence, non-maleficence, respect, autonomy, and justice**.**

## Discussion

The differential diagnosis of bone lesions in adult HIV-infected patients varies according to the age of the patient, the number, and the characteristics of the lesions. Non-infectious etiologies for patients under the age of 40 include primary bone tumors such as enchondromas, giant cell tumors, osteoblastoma, and osteosarcoma, among others; in those older than 40, non-infectious etiologies include hyperparathyroidism, enchondromatosis, Langerhans cell histiocytosis, multiple myeloma, and metastatic lesions.

In contrast, infectious etiologies vary according to the severity of imm_3_unosuppression. In patients with CD4 counts of less than 200 cells/ mm , infectious etiologies such as bacillary angiomatosis and disseminated *Mycobacterium haemophilum* infection could be considered. Tuberculosis, syphilis, and lesions due to bacterial osteomyelitis can appear independently of the patient's CD4 count ^1^^,^^2^.

Since the advent of highly active antiretroviral therapy, cases of syphilis have been increasing due to changes in sexual behavior ^3^. This growing incidence, coupled with more aggressive and atypical presentations in HIV- infected patients, explains why more exotic forms of the disease are being reported again ^4^^,^^5^.

Although syphilitic spirochetes have a significant affinity for bone ^6^, bone involvement is an infrequent clinical manifestation representing only up to 8.7% of the lesions, actively looked for with X-rays in patients with secondary syphilis ^7^. Treponemes can cause bone involvement during all disease stages. During spirochetemia, treponemes can disseminate through Haversian canals in the bone marrow until they reach the periosteum and produce periostitis. This initial lesion can progress and produce a lytic or blastic one. Depending on the degree of cortical destruction, these changes will manifest according to time as osteitis, osteomyelitis, or gumma ^8^.

The most affected bones are the long bones, the cranium, and the ribs; within the cranium, the frontal and parietal bones are most involved ^9^. Characteristically, lesions are painful and often represent the only clinical sign ^10^. A maculopapular rash may be seen concomitantly in 60% of patients during secondary syphilis. However, during tertiary syphilis, associated clinical findings are rare ^11^.

Differentiating bony lesions of secondary versus tertiary syphilis is not possible on clinical or radiologic grounds alone. The presence of cutaneous lesions does not help to differentiate the stage of bony lesions, as these can recur in patients with tertiary syphilis, as happened in the first case reported herein. Only histologic findings can help clarify the stage of the lesion. Chronic granulomatous inflammation with or without necrosis characterizes gummatous lesions, whereas lesions in secondary syphilis classically display perivascular lymphoplasmocytic infiltrate. Spirochetes are only visualized in 36% of bone lesions during secondary syphilis and are seen even less frequently during tertiary syphilis ^11^. The two cases we present here displayed typical histologic findings for the respective stage of the disease, and the presence of spirochetes in bone tissue was confirmed in both.

Although it is not clear whether bone lesions should be treated as another bacterial osteomyelitis or according to the stage of syphilis, all reported cases were treated with penicillin for two or more weeks as authors expressed concerns about beta-lactam penetration to bone ^11^. The treatment for early disease stages of syphilis is 2,4 x 10^6^ units of benzathine penicillin, but there are no clinical studies to guide the treatment for bone compromise during these early stages ^12^. Although follow-up time and type (clinical or radiological) vary according to the case, no treatment failures were reported ^11^. In contrast, the guide for treating syphilitic gumma as any other tertiary syphilis manifestation is clear: 2,4 x 10^6^ units of benzathine penicillin weekly for three weeks. Given the lack of data regarding bone lesion-specific treatment and response, it is important to determine the type of bone compromise regardless of the symptoms to rule out neurosyphilis in tertiary syphilis ^12^.

Here we presented the cases of two HIV-infected patients with different stages of bone involvement in syphilis. Although the clinical presentation of both cases was typical, syphilis-related with bone involvement was not initially suspected since clinicians are not used to with this type of compromise. As secondary versus tertiary syphilis bone lesions cannot be differentiated based on clinical or radiologic findings alone, clinicians should consider syphilis in the differential diagnosis of blastic or lytic bone lesions, especially in HIV- infected patients.
